# Lymph nodes tuberculosis: a retrospective study on clinical and therapeutic features

**DOI:** 10.11604/pamj.2015.20.65.5782

**Published:** 2015-01-23

**Authors:** Amine Benjelloun, Youssef Darouassi, Yasser Zakaria, Rachid Bouchentouf, Noureddine Errami

**Affiliations:** 1 Pulmonology Unit, Avicenne Military Hospital, Marrakech, Morocco; 2 ENT Unit, Avicenne Military Hospital, Marrakech, Morocco; 3 ENT Unit, Mohammed V Military Hospital, Rabat, Morocco

**Keywords:** Lymph nodes, tuberculosis, extra pulmonaty

## Abstract

Lymph nodes tuberculosis represents 30 percent of extra pulmonary tuberculosis in Morocco. We report here the experience of the pulmonology unit of the Avicenne Military Hospital in Marrakech for a period of 4 years. Our study interested 30 patients (15 males and 15 females) with an average age of 29 years old (10 to 62 years old). Tuberculosis has interested a single site in 28 patients, the other two patients had multiple but unilateral involvement. For the single sites, locations were jugulo-carotidian (20 patients), supra-calvicular (2 patients), axillary (2 patients), sub-mandibular (2 patients), spinal (2 patients) and mediastinal (2 patients). For the multiple locations, the involvement was jugulo-carotidian, sub-mandibular, spinal and sub-clavicular for one patient; and jugulo-carotidian and sub-clavicular for the other. Diagnosis was made by surgical biopsy and histology for all the patients. A six-month anti-tuberculous treatment was given afterward, with relapses in two patients.

## Introduction

Tuberculosis remains a real public health problem in Morocco with an average incidence of 26 000 new cases per year. Extra pulmonary tuberculosis (EPT) represents 46% of all cases. Lymph node and pleural tuberculoses are the most common forms of EPT [1].

## Methods

The study purpose was to analyze the epidemiological and evolutionary profile of lymph node tuberculosis in our service after histological confirmation and six-month anti-tuberculous treatment. This is a retrospective study of 30 patients with lymph node tuberculosis followed in our unit over a period of 4 years.

## Results

Table 1

The study concerned 15 women and 15 men with an average age of 29 years old (range was 10 to 62 years old). Tuberculosis has interested a single site in 28 patients, the other two patients had multiple but unilateral involvement. For the single sites, locations were jugulo-carotidian (20 patients), supra-calvicular (2 patients), axillary (2 patients), sub-mandibular (2 patients), spinal (2 patients) and mediastinal (2 patients). For the multiple locations, the involvement was jugulo-carotidian, sub-mandibular, spinal and sub-clavicular for one patient; and jugulo-carotidian and sub-clavicular for the other. One patient had diabetes. None of the patients was HIV positive. General signs, such as asthenia, sweats, fever, and weight loss were found in 4 patients (13%). No immune-suppression was noticed. The diagnosis was obtained by surgical biopsy and histological analysis in all patients. For the two patients with mediastinal tuberculosis, diagnosis was respectively obtained by thoracotomy and mediastinoscopy. A chest radiograph was systematically performed for all patients looking for other locations. One patient (sub-Saharan origin) had an associated pleurisy. The patients were given a six-month anti-tuberculous treatment: 2RHZE/4RH (R:Rifampicin 10 mg/ kg/ j, H:Isoniazid 5 mg/ kg/ j, Z:Pyrazinamid 25 mg/ kg/j) with an uneventful evolution for 28 patients. One patient had an ipsilateral relapse healed with an eight-month treatment. Another woman relapsed twice despite prolonged treatment.

**Table 1:** Clinical and therapeutic patients features

Patients (N = 30)	Number	Percentage
Mean age	29 years (10 to 62 years)	
**Sex**		
Male	15	50%
Female	15	50%
**Risk factors**		
HIV	None	
Diabetes	1	3.3%
**Localisations**		
Jugulo-carotidian	20	66%
Supra-clavicular	2	6.6%
Sub-mandibular	2	6.6%
Spinal	2	6.6%
Mediastinal	2	6.6%
Multiple	2	6.6%
**Associated locations**		
Lung	None	
Pleura	1	3.3%
**Relapse**	2	6.6%
**Treatment duration**		
6 months (first hand)	30	100%
8 months (relapse)	2	6.6%

## Discussion

Lymph node tuberculosis represents 30% of extra pulmonary tuberculosis in Morocco, 25% in France and 16.7% in the USA, with predominance of cervical forms (80% of our patients) [1, 2]. As in our series, it usually affects children and young adults between 20 and 40 years old [3]. The disease is less common in Caucasians and women, while the gender ratio is 1:1 in our series. The disease is usually favoured by promiscuity, immune deficit, HIV and diabetes [4, 5]. General signs (weight loss, sweats, fever, and asthenia) are found in 20 to 50% [6, 7]. The lymph node TB usually causes a painful swelling of one or more lymph nodes. Most often, the disease is localized to the anterior or posterior cervical chains (70-90%) or supra clavicular. It is often bilateral and non-contiguous lymph nodes can be involved [3]. The jugulo-carotidian location is the most common and relapses occur in about 5% of cases (6.6% in our series) [8]. The overlying skin is first normal, but changes gradually until fistulisation. This fistula heals with difficulty. The diagnosis of lymph node tuberculosis can be established by puncture of the lymphadenopathy upper pole for direct microbiological study and culture. This examination is only positive in 30% of all cases [8]. PCR analysis of the node sample may be of great help for a quick diagnosis.

When the bacillus is not found, histology may help by showing an epithelioid and giganto cellular granuloma with caseous necrosis in immunocompetent patients. Surgical biopsy-resection of the lymph node is the best examination for diagnostic confirmation, with a sensitivity of 100% for histological analysis and 60-90% for the bacilli culture [7, 9]. In our series, the diagnosis was always histological. The biopsy may be preceded by a spotting neck ultrasound. A chest radiograph must always be performed to look for other locations of tuberculosis. Some authors also advocate systematic abdomen ultrasound. A six-month anti tuberculous regimen is usually sufficient for healing. This treatment should not be undertaken without histological or bacteriological confirmation because of possible associated hemopathy [8]. Treatment of lymph node tuberculosis has much evolved in the past decade. Many practitioners used to give nine, twelve sometimes eighteen months regimen, thinking lymph node penetration of antibiotics is insufficient. Actually, the WHO protocol emphasizes that a six-month regimen is enough [1]. Sometimes, the lymph nodes can increase in number (48%) and/or in volume (36%), a few weeks after the beginning of treatment without pathological significance [10]. Place of surgery is controversial but becomes essential in case of residual lymph nodes, relapses and giant abscesses.

## Conclusion

Lymph node tuberculosis is common in North Africa as well as other locations. However histological confirmation should always be obtained before starting treatment even in the presence of pulmonary tuberculosis, to avoid missing an associated hemopathy.

## References

[1] (2011). Programme National de lutte antituberculeuse (Maroc).

[2] Nong BR, Chuang CM, Huang YF (2009). Ten-years experience of children with tuberculosis in southern Taiwan. J microbiol Immunol Infect..

[3] Arstenstein AW, Kim JH, Williams WJ, Chung CY (1995). Isolated peripheral tuberculous lymphadenitisin adults: current clinical and diagnosis issues. Clin Infect Dis..

[4] Ilgazli A, Boyaci H, Basyigit I, Yildiz F (2004). Extra pulmo-nary tuberculosis: clinical and epidemiologic spectrum of 636 cases. Arch Med Res..

[5] Kim SJ, Hong YP, Lew WJ (1995). Incidence of pulmonary tuberculosis among diabetics. Tuber Lung Dis..

[6] Hochedez P, Zeller V, Truffot C (2003). Lymph-node tuberculosis in patients infected or not with HIV: general characteristics, clinical presentation, microbiological diagnosis and treatment. Pathologie Biologie..

[7] Lacut JY, Dupon M, Pathy MC (1995). Tuberculoses extra-pulmonaires: revue et possibilités de diminution des délais d'intervention thérapeutique. Med Mal Infect..

[8] Elloumi M, Fakhfakh S, Frikha M (1999). Aspects diagnostique et thérapeutique de la tuberculose ganglionnaire: à propos de 41 cas. La Tunisie médicale..

[9] Ammari FF, Bani Hani AH, Ghariebeh KI (2003). Tuberculosis of the lymph glands of the neck: a limited place for surgery. Otolaryngol Head Neck Surg..

[10] British Thoracic society Research comittee (1985). Sort course chemotherapy for tuberculosis of lymph nodes: a controlled trial. Br Med J..

